# Safe Synthesis of Alkylhydroxy and Alkylamino Nitramines

**DOI:** 10.3390/molecules21121738

**Published:** 2016-12-16

**Authors:** Simen Antonsen, Marius Aursnes, Harrison Gallantree-Smith, Christian Dye, Yngve Stenstrøm

**Affiliations:** 1 Department of Chemistry, Biotechnology and Food Science, Norwegian University of Life Sciences, P.O. Box 5003, NO-1433 Ås, Norway; simen.antonsen@nmbu.no (S.A.); marius.aursnes@farmasi.uio.no (M.A.); harrison.gallantree-smith@nmbu.no (H.G.-S.); 2 Institute for Energy Technology (IFE), NO-2027 Kjeller, Norway; Christian.Dye@ife.no; 3 Norwegian Institute for Air Research (NILU), NO-2027 Kjeller, Norway

**Keywords:** nitramines, safe method, carbon capture, AMP, MEA

## Abstract

Three different protocols for the syntheses of hydroxyalkylnitramines are presented and compared. Safety issues regarding the synthesis of nitramines are also discussed.

## 1. Introduction

Carbon capture of today has been implemented as an important tool for controlling the greenhouse effect on our planet. Several technologies for carbon capture and storage (CCS) have been developed, but amine-based technologies have been found to be the most promising [1]; especially the aminoalkanols, such as 2-amino-1-ethanol (MEA; **1**) and 2-amino-2-methyl-1-propanol (AMP; **2**) [2]. The use of MEA for such a purpose has been implemented on a large scale by Statoil in the North Sea, since the mid-1990s [3]. One major drawback associated with the amine-based CCS is the formation of harmful byproducts: i.e., the reaction of the amino group to give nitramines and nitrosamines [4]. This has spurred a new interest in these compounds [5,6,7,8,9,10]. A recent review summarizes the hazard assessment of these as byproducts of amine-based CCS [7]: nitramines are less carcinogenic than the corresponding nitrosamine [11] and formed in lower concentration in CO_2_ capture systems [3,12], but they are more persistent in the environment due to the lack of sunlight photolysis [9,13]. However, the conclusion is that more studies are needed.

Although nitramines have been known for a long time, they have been made mostly for the purpose of making explosives (e.g., 1,3,5-trinitroperhydro-1,3,5-triazine (RDX), Octahydro-1,3,5,7-tetranitro-1,3,5,7-tetrazocine (HMX), nitroguanidine and tetryl) and rocket fuels [14,15]. On the other hand, their use in synthesis has been exploited only to a very limited extent. The best example of this is the use of primary nitramines in the Mannich reaction [16].

To learn more about these byproducts, we started a new project aiming for a practical and safe synthesis of pure nitramines. The general method for their syntheses has been to add 70%–100% nitric acid to the corresponding amine. This is a general and fairly efficient method. However, several problems may be encountered with this procedure, such as a lack of chemoselectivity. The use of the obnoxious and highly corrosive concentrated or pure nitric acid is by itself a hazard. Special precautions must be taken to avoid over-nitration, which will pose an increased risk for explosions [17].

One way of avoiding the nitration of the alcohol would, of course, be protection. A simple and efficient protection method for an alcohol is transformation to an ester, usually by adding acetyl chloride or acetic anhydride. However, most of these methods will also give the corresponding amide when amino groups are present, primary amines being especially reactive. This will immediately pose two problems: the amide will be less reactive than the amine, and fairly harsh conditions are needed for the deprotection of both the ester and nitrated amide.

The challenge will be to find protection groups that are both stable to the nitration conditions and should either be chemoselective (i.e., not react with the amine) or the protected amine would have to be reactive towards the nitration agent.

Furthermore, the protection group should be removable without affecting the nitramine and to avoid the formation of metal salts of the nitramines, which are quite acidic (pK_a_ ≈ 6). Metal salts of these are known to be even more prone to detonation, and as such, special precautions have to be taken before handling them [18].

## 2. Results and Discussion

### 2.1. MEA Nitramine

**Scheme 1:**
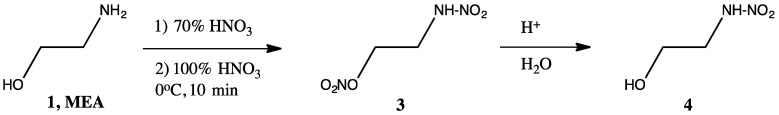
Possible synthesis of 2-hydroxyethylnitramine [20].

The only synthesis of MEA nitramine (**4**) described in the literature is a patented procedure in which nitramide is reacted with ethylene oxide [19]. Other than this, the compound is mentioned briefly as an intermediate in a paper from 1953, though no isolation of the compound was reported [20]. The authors made dinitrated MEA followed by hydrolysis and reported that the oxygen-nitro group is hydrolyzed more easily than the nitrogen (Scheme 1).

Although this method is an option, the two major drawbacks are (1) the ability to stop the hydrolysis in due time before full hydrolysis occurs as nitramines are unstable in acidic water solutions and, more severely; (2) the dinitrated compound, 2-nitroaminoethyl nitrate, is expected to be even more prone to explosion. However, it was anticipated that the protected MEA, e.g., 2-aminoethyl acetate, could be used; though the ester may hydrolyze under the given nitrating conditions, giving a possibility for the alcohol to be deprotected and nitrated, as well. Although this should be possible to handle by monitoring the hydrolysis reaction, it will still pose a risk.

**Scheme 2:**
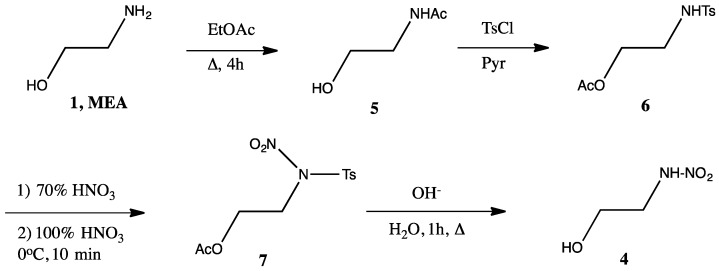
Attempted synthesis of 2-hydroxyethylnitramine.

Another variety of this strategy would be to prepare the nitramines from the corresponding tosylamides [21]. This has worked well in our laboratory, and we decided to try the same strategy for MEA. The acetate of 2-((4-methylphenyl)sulfonamido)ethyl (**5**) can be prepared using a literature procedure [22]. The strategy is depicted in Scheme 2.

At first, we performed nitration and hydrolysis in one batch without isolating the nitro tosylate intermediate followed by exclusion of nitric acid prior to hydrolysis, but all attempts to do so failed, resulting only in impure samples containing less than 10% of the title compounds.

Therefore, we decided to isolate the nitro tosylatamide **7**. The classic way to make nitramines is to react an amine with a huge excess of fuming nitric acid, followed by evaporation to dryness on a steam bath [23]. Following this procedure, we managed to obtain the nitro tosylamide **7** in a 70% yield, but only in an inadequate purity. We decided to try evaporation assisted by a flow of nitrogen gas at room temperature. However, it was not possible to achieve reproducible results comparable to the previous experiments; neither conversion, yield nor purity could be reproduced. Production of obnoxious and corrosive nitrous gases is also a severe disadvantage of such a method.

Different hydrolytic conditions were also attempted, but as this strategy depended on basic hydrolysis, inorganic salts will be present. At best, the NMR analysis implied a mixture consisting of some 70% of the desired compound. Therefore, this strategy was eventually abandoned.

**Scheme 3:**
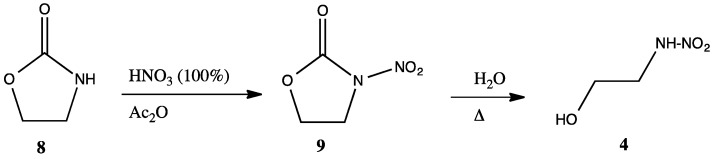
Synthesis of 2-hydroxyethylnitramine.

We still figured some kind of protection would be the best way of synthesizing 2-hydroxyethylnitramine. Starting with commercially-available 2-oxazolidone (**8**) should be ideal, since hydrolysis of the nitrated compound should give 2-hydroxyethylnitramine (**4**) with carbonic acid as the only side product. Therefore, we set forth this reaction as depicted in Scheme 3.

The nitration method described above worked out beautifully. Nitramine **9** was obtained as pure after recrystallization with ethanol. We tried to do the hydrolysis in neutral water, and with slight heating, this also worked out smoothly. The product was isolated as the pure compound. It crystallizes at low temperature and can also be recrystallized this way. However, since it is found that the nitrated oxazolidone is much easier to handle, being a high-melting crystalline compound, it is better to stock the compound as such and then hydrolyze it when needed.

However, evaporation of fuming nitric acid as an obnoxious gas is not feasible. Therefore, we were looking for an improvement. One known alternative is the combination of acetic anhydride and nitric acid at low temperature, to give AcO-NO_2_, which is known as an effective nitration agent [24]. We used a slight modification on the published procedures by White et al. [25]. The evaporation of fuming nitric acid will henceforth be referred to as Method A, while the latter, employing AcO-NO_2_, will be referred to as Method B.

Another alternative is employing copper nitrate in combination with acetic anhydride. We found this method preferable; as it does not use the corrosive fuming nitric acid at all [26]. This method will be referred to as Method C.

All three nitration methods gave the compound in good yield, although Method A gave a slightly better yield than the two other ones.

### 2.2. AMP Nitramine

Strategies similar to the ones illustrated in Scheme 1 and Scheme 2 were used on AMP with the same result. With the success making 2-hydroxyethylnitramine via the oxazolidone, we thought the same method would apply to AMP.

**Scheme 4:**
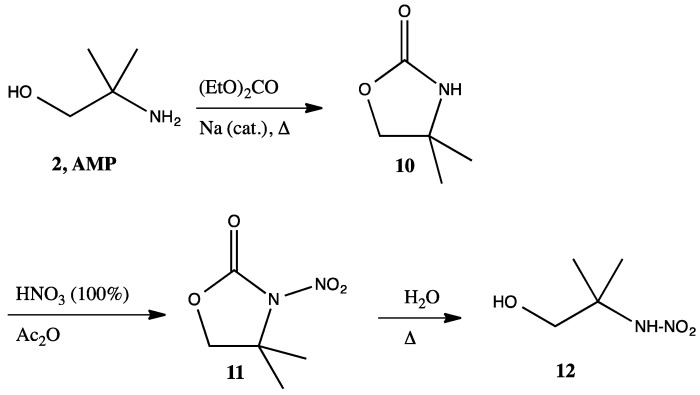
Synthesis of 1-hydroxy-2-methylpropan-2-ylnitramine.

Using a modified literature procedure starting from 2-amino-2-methyl-1-propanol (**2**), we made the corresponding oxazolidone **9** on a large scale in a very good yield [25]. See Scheme 4.

The same protocols for nitration were used. Method A resulted in a complex mixture of products, and purification gave a drastic drop in the isolated yield, while both Method B and C gave Product **11** in high yields (Table 1, Entry 2), which was easily purified by recrystallization.

Hydrolysis of the carbamate using the neutral conditions mentioned above did indeed work excellently. Again, we figured that due to the decreased stability of the final, open chain nitramine, it would be more practical to handle the high melting crystalline heterocycle and do the hydrolysis only when the nitramine was needed.

### 2.3. Scope and Limitations

**Figure 1:**
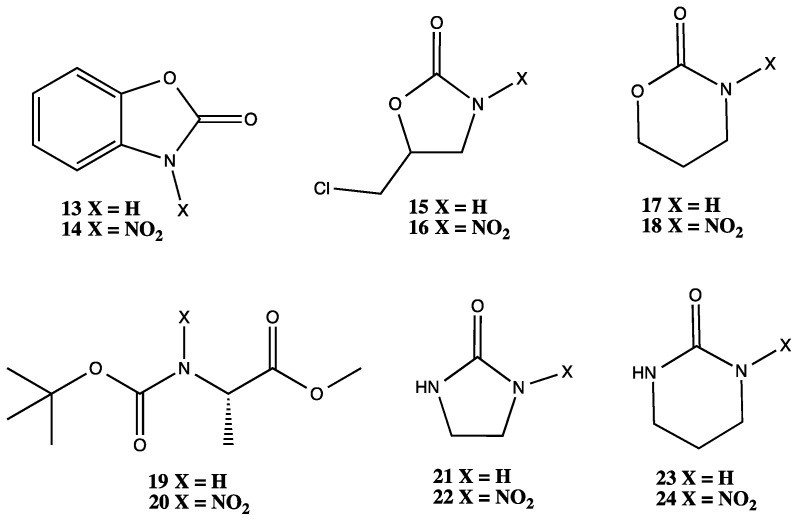
Structure of carbamates and cyclic urea derivatives.

In order to explore the scope and limitations of Method C vs. Methods A and B, we tested all three methods on the carbamates. The structures are shown in Figure 1, and the results are listed in Table 1.

For benzo[*d*]oxazol-2(3*H*)-one (**13**), both reactions using nitric acid gave a lower yield of nitramine **14** compared to the one employing Cu(NO_3_)_2_. This can be explained by the fact that we had purification problems related to the removal of nitric acid. Somehow, we had problems removing all of the nitric acid, and when heated, the mixture exploded, possibly due to nitration of the aromatic ring.

For 5-(chloromethyl)-oxazolidin-2-one (**15**), Method C gave a lower yield of nitramine **16** compared to Method A and B. This is probably due to some competitive reaction with the halogen and copper in Method C.

Nitration of 1,3-oxazinan-2-one (**17**) gave approximately the same yield of nitramine **18** with all three methods.

We know that the non-cyclic carbamates, e.g., Boc-protected amines, will be cleaved when treated with strong acids (e.g., HCl or CF_3_CO_2_H), and we were expecting the same thing to happen under the conditions of Method B and C. That was also what we observed when methyl(*tert*-butoxycarbonyl)alaninate (**19**) was attempted to be nitrated. The reactions led to complex mixtures, and the desired nitramine **20** was not observed at all. Conversely, using Method C gave the desired nitramine in an acceptable yield, as listed in Table 1, Entry 6.

To extend the potential of the reaction, we also tried to mononitrate the bisamines using the corresponding cyclic urea derivatives. Obviously, the number of equivalents had to be changed to avoid over nitration. Method A was abandoned, since it was anticipated to give a mixture of mono- and di-substituted products together with unreacted material. A preliminary experiment confirmed this. Method B gave the mononitramine of structure imidazolidin-2-one (**22**) and tetrahydropyrimidin-2(1*H*)-one (**24**) in yields of 60% and 70%, respectively. However, it was discovered that a published synthesis of 1-nitroimidazolidin-2-one uses similar conditions, but at room temperature in a shorter reaction time [27]. Employing the already published conditions, we synthesized 1-nitrotetrahydropyrimidin-2(1*H*)-one in a good yield (70%).

Method **C** was tested on the same urea derivatives, but the yield was extremely poor. Increasing the amount of Cu(NO_3_)_2_ gave a mixture of mono- and di-substituted products together with unreacted material.

## 3. Materials and Methods

General: All reagents and solvents were commercial grade and used without further purification. The NMR spectra were recorded on a Varian Gemini 300 (Varian Inc., Palo Alto, CA, USA) or a Bruker AVANCE 400 spectrometer (Bruker Corporation, Billerica, MA, USA). IR spectra were obtained on a reflectance cell (HATR) on a Perkin Elmer FTIR instrument (PerkinElmer, Inc., Waltham, MA, USA). High resolution mass spectra were obtained for 2-hydroxyethylnitramine and 1-hydroxy-2-methylpropan-2-ylnitramine using an Agilent 1100 liquid chromatography system (Agilent Technologies, Waldbronn, Germany), equipped with an Agilent 1100 auto-sampler, an Agilent 1100 quaternary pump, an Agilent 1100 on-line degassing system and an Agilent 1100 diode array detector (UV). The analytical detector was a Micromass LCT orthogonal-acceleration time-of-flight (TOF) mass spectrometer (MS) equipped with a Z-spray electrospray ion source and a 4-GHz time to digital converter (TDC) (Waters Corporation, Milford, MA, USA). NMR and IR spectra can be found in “Supplementary Materials”.

In particular handling of concentrated and fuming nitric acid (HNO_3_) needs special precautions and safety equipment. Furthermore, all nitrated compounds should be treated as potential explosives.

### 3.1. General Nitration Procedures

#### 3.1.1. Method A

The carbamate (115 mmol) was added portion wise to stirred nitric acid (100%; 28 g; 444 mmol). The solution was evaporated to dryness on a steam bath. The product solidified on cooling; it was dried in vacuo and recrystallized from toluene (absolute. ethanol can also be used) to give the product.

#### 3.1.2. Method B

Using a dry ice/acetone bath acetic anhydride (25 mL) was cooled to −15 °C. Fuming nitric acid (18.5 g; 13.2 mL; 298 mmol) was added dropwise keeping the temperature between −10 and −15 °C. A mixture of the carbamate (24.0 mmol) and about 12.5 mL of Ac_2_O was added slowly at −15 °C with vigorous stirring. The mixture was allowed to warm up to 0 °C and maintained at 0–4 °C for 10 h. The reaction mixture was poured on ice and stirred for a couple of minutes. Extraction with DCM, washing with 10% NaHCO_3_, drying (MgSO_4_) and evaporation yielded the product.

#### 3.1.3. Method C

A solution of the carbamate (115 mmol) in 72 mL Ac_2_O was added slowly with stirring to a cooled (5–10 °C) suspension of finely-ground Cu(NO_3_)_2_·(H_2_O)_3_ (10.5 g; 43 mmol) in 85 mL Ac_2_O. After 1 h, an additional portion of finely-ground Cu(NO_3_)_2_·(H_2_O)_3_ (10.5 g; 43 mmol) was added. The resulting mixture was cooled to 0–5 °C (ice/water) and stirred for another 4 h while slowly being heated to room temperature. The mixture was filtered through Celite 545. Water (50 mL) was added, and the water phase was extracted with DCM (3 × 50 mL) to get rid of additional dissolved copper salts. Drying (MgSO_4_) and evaporation gave a crystalline compound that was recrystallized from toluene to give the product.

### 3.2. Spectral Data

*3-Nitrooxazolidin-2-one* (**9**): Oxazolidin-2-one (**8**) was nitrated using Methods A, B and C to give **9** as off-white crystals. m.p. 109–110 °C (literature.^1^ m.p. 108–109.5 °C). ^13^C-NMR (100 MHz, CDCl_3_): δ 45.22, 60.18, 147.45. ^1^H-NMR (400 MHz, CDCl_3_): δ 4.39 (2H, d, *J* = 7.2 Hz), 4.32 (2H, *J* = 7.2 Hz). IR: 3006, 1794, 1565 cm^−1^.

*4,4-Dimethyl-3-nitrooxazolidin-2-one* (**11**): 4,4-dimethyloxazolidin-2-one (**10**) was nitrated using Methods A, B and C to give **11** as white crystals. m.p. 121–122 °C (lit.^1^ m.p. 124 °C). ^13^C-NMR (100 MHz, CDCl_3_): δ 22.02, 62.92, 72.18, 147.65. ^1^H-NMR (400 MHz, CDCl_3_): δ 4.11 (6H, s), 1.68 (2H, s). IR: 3005, 2992, 2949, 1772, 1565 cm^−1^.

*3-Nitrobenzo[d]oxazol-2(3H)-one* (**14**): Benzo[*d*]oxazol-2(3*H*)-one (**13**) was nitrated using Methods A, B and C to give **19** as white crystals, slightly yellow. m.p. was not determined, as the compound decomposed on the apparatus. ^13^C-NMR (100 MHz, CD_3_OD): δ 109.32, 114.93, 130.11, 132.73, 141.52, 145.38, 153.99. ^1^H-NMR (400 MHz, CD_3_OD): δ 8.44 (2H, d, *J* =1.8 Hz), 8.86 (2H, d, *J* = 1.8 Hz). IR: 3103, 1798, 1637, 1543 cm^−1^.

*5-(Chloromethyl)-3-nitrooxazolidin-2-one* (**16**): 5-(chloromethyl)oxazolidin-2-one (**15**) was nitrated using Methods A, B and C to give **21** as white crystals. m.p. was not determined, as the compound decomposed on the apparatus. ^13^C-NMR (100 MHz, CDCl_3_): δ 42.77, 47.50, 70.14, 146.45. ^1^H-NMR (400 MHz, CDCl_3_): δ 3.69–3.80 (2H, m), 4.25 (1H, dd, *J* = 5.9 Hz, *J* = 4.3 Hz), 4,42 (dd, *J* = 8.5 Hz, *J* = 4.3 Hz), 4.81–4.87 (1H, m). IR: 3025, 2968, 2929, 2853, 1804, 1571 cm^−1^.

*3-Nitro-1,3-oxazinan-2-one* (**18**): 1,3-oxazinan-2-one (**17**) was nitrated using Methods A, B and C to give **21** as off-white crystals. m.p. 75–76 °C (lit.^1^ 74 °C). ^13^C-NMR (100 MHz, CDCl_3_): δ 22.56, 47.77, 66.88, 146.52. ^1^H-NMR (400 MHz, CDCl_3_): δ 2.24 (2H, m), 4.12 (2H, t, *J* = 6.7 Hz), 2.24 (2H, t, *J* = 5.5 Hz). IR: 1802, 1572 cm^−1^.

*Methyl N-(tert-butoxycarbonyl)-N-nitro-alaninate* (**20**): Methyl *N*-(tert-butoxycarbonyl)-alaninate (**19**) was nitrated using Method C to give 22 as a white/clear oil. ^13^C-NMR (100 MHz, CDCl_3_): δ 14.16, 27.72, 52.77, 56.64, 86.65, 148.67, 169.20. ^1^H-NMR (400 MHz, CDCl_3_): δ 1.55 (9H, s), 1.61 (3H, d, *J* = 7.0 Hz), 3.76 (3H, s), 5.22 (1H, q, *J* = 7.0 Hz). IR: 2986, 1749, 1582.

*1-Nitrotetrahydropyrimidin-2(1H)-one* (**24**): Tetrahydropyrimidin-2(1*H*)-one (**23**) was nitrated a modification of Methods B, as described by Astakhov et al. [20], to give **23** as white crystals. ^13^C-NMR (100 MHz, CDCl_3_): δ 150.28, 22.49., 39.84, 48.20. ^1^H-NMR (400 MHz, CDCl_3_): δ 6.37 (1H, bs), 4.12 (2H, t, *J* = 6.2 Hz), 3.37 (2H, m), 2.17 (2H, q, *J* = 6.2 Hz). IR: 1694, 1532 cm^−1^.

## 4. Conclusions

We have tested and compared three methods for the nitration of amino alcohols. Method C has proven to be a reliable and safe alternative to the classic Methods A and B when an excess of the NO_2_^+^-source can be tolerated. Nitration of more sensitive compounds (e.g., Boc-protected alanine ethyl ester) was also possible with this protocol, but mono-nitration on diamines does not work.

## Figures and Tables

**Table 1:** Reaction yield for the different nitration methods on the carbamates compared.

Entry	Starting Material	Yields (%)			Product
		A	B	C	
1	2-oxazolidone (**9**)	78	65	59	**4**
2	4,4-dimethyloxazolidin-2-one (**10**)	43	63	62	**11**
3	benzo[*d*]oxazol-2(3*H*)-one (**13**)	54	32	74	**14**
4	5-(chloromethyl)-oxazolidin-2-one (**15**)	69	70	54	**16**
5	1,3-oxazinan-2-one (**17**)	56	59	62	**18**
6	Methyl (*tert*-butoxycarbonyl)alaninate (**19**)	- ^a^	- ^a^	52	**20**

Yields are listed for pure material, i.e., after recrystallization. ^a^ Resulted in a complex mixture, with no traces of desired product according to NMR.

## Supplementary Materials

Supplementary materials can be accessed at: http://www.mdpi.com/1420-3049/21/12/1738/s1.

## References

[1] Walters M.S., Edgar T.F., Rochelle G.T. (2016). Regulatory Control of Amine Scrubbing for CO_2_ Capture from Power Plants. Ind. Eng. Chem. Res..

[2] Rochelle G.T. (2009). Amine Scrubbing for CO_2_ Capture. Science.

[3] Dai N., Shah A.D., Hu L., Plewa M.J., McKague B., Mitch W.A. (2012). Measurement of Nitrosamine and Nitramine Formation from NOx Reactions with Amines during Amine-Based Carbon Dioxide Capture for Postcombustion Carbon Sequestration. Environ. Sci. Technol..

[4] Rochelle G.T., Fine N.A. (2014). Absorption of Nitrogen Oxides in Aqueous Amines. Energy Procedia.

[5] Helgesen L.I., Gjernes E. (2016). A way of qualifying Amine Based Capture Technologies with respect to Health and Environmental Properties. Energy Procedia.

[6] Coutris C., Macken A.L., Collins A.R., Yamani N., Brooks S.J. (2015). Marine ecotoxicity of nitramines, transformation products of amine-based carbon capture technology. Sci. Total Environ..

[7] Buist H.E., Devito S., Goldbohm R.A., Stierum R.H., Venhorst J., Kroese E.D. (2015). Hazard assessment of nitrosamine and nitramine by-products of amine-based CCS: Alternative approaches. Regul. Toxicol. Pharmacol..

[8] Gundersen C.B., Andersen T., Lindahl S., Linke D., Vogt R.D. (2014). Bacterial response from exposure to selected aliphatic nitramines. Energy Procedia.

[9] Nielsen C.J., Herrmann H., Weller C. (2012). Atmospheric chemistry and environmental impact of the use of amines in carbon capture and storage (CCS). Chem. Soc. Rev..

[10] Nielsen C.J., D’Anna B., Dye C., Graus M., Karl M., King S., Maguto M.M., Müller M., Schmidbauer N., Stenstrøm Y. (2001). Atmospheric chemistry of 2-aminoethanol (MEA). Energy Procedia.

[11] Wagner E.D., Osiol J., Mitch W.A., Plewa M.J. (2014). Comparative in vitro toxicity of nitrosamines and nitramines associated with aminebased carbon capture and storage. Environ. Sci. Technol..

[12] Dai N., Mitch W.A. (2014). Effects of flue gas compositions on nitrosamine and nitramine formation in postcombustion CO_2_ capture systems. Environ. Sci. Technol..

[13] Pitts J.N., Grosjean D., Vancauwenberghe K., Schmid J.P., Fitz D.R. (1978). Photo-oxidation of aliphatic-amines under simulated atmospheric conditions–formation of nitrosamines, nitramines, amides, and photo-chemical oxidant. Environ. Sci. Technol..

[14] Beckstead M.W., Puduppakkam K., Thakre P., Yang V. (2007). Modeling of combustion and ignition of solid-propellant ingredients. Prog. Energy Combust. Sci..

[15] Sikder A.K., Maddala G., Agrawal J.P., Singh H. (2001). Important aspects of behaviour of organic energetic compounds: A review. J. Hazard. Mater..

[16] Buckle D.R., Pinto I.L., Katritzky A.R., Meth-Cohn O., Rees C.W. (1995). 4.09—Functions Bearing Two Nitrogens. Comprehensive Organic Functional Group Transformations.

[17] Agrawal J.P., Hodgson R.D. (2007). Organic Chemistry of Explosives.

[18] Matyáš R., Pachman J. (2013). Primary Explosives.

[19] Jianming Y., Lv J., Yu Q., Li F., Liu B., Wang W. (2011). Method for Preparing N-Nitroamino Alcohol.

[20] Barrott J., Denton I.N., Lamberton A.H. (1953). Nitramines and nitramides. Part IV. The acid-catalysed decomposition of primary nitramines. J. Chem. Soc..

[21] Yamaguchi T., Hesek D., Lee M., Oliver A.G., Mobashery S. (2010). Sulfonylation-induced N- to O-acetyl migration in 2-acetamidoethanol derivatives. J. Org. Chem..

[22] Maguta M.M., Aursnes M., Bunkan A.J.C., Edelen K., Mikoviny T., Nielsen C.J., Stenstrom Y., Tang Y., Wisthaler A. (2014). Atmospheric fate of nitramines: An experimental and theoretical study of the OH reactions with CH_3_NHNO_2_ and (CH_3_)_2_NNO_2_. J. Phys. Chem..

[23] Franchimont A.P.N., Lublin A. (1902). Nitroamino-alcohols. Rec. Trav. Chim..

[24] Bordwell F.G., Garbisch E.W. (1960). Nitrations with acetyl nitrate. I. The nature of the nitrating agent and the mechanism of reaction with simple alkenes. J. Am. Chem. Soc..

[25] White E.H., Chen M.C., Dolak L.A. (1966). *N*-nitroamides and *N*-nitrocarbamates. III. Rotational isomerism, steric effects, and physical properties at low temperatures. J. Org. Chem..

[26] Menke J.B. (1925). Nitration with nitrates. Recl. Travaux Chim. Pays-Bas.

[27] Astakhov M., Stepanov R.S., Kruglyakova L.A., Kekin Y.V. (2000). 1-Nitroimidazolidin-2-one and its hydrolysis to 1-amino-2-nitroaminoethane. Russ. J. Org. Chem..

