# Supplementary material for: Safe Synthesis of Alkylhydroxy and Alkylamino Nitramines
Source: Molecules. 2016 Dec 16;21(12):1738. doi: 10.3390/molecules21121738 (PMC6274293; doi:10.3390/molecules21121738)
Supplement: Supplementary file 1 [file molecules-21-01738-s001.pdf]

Supplementary Materials: Safe Synthesis of Alkylhydroxy and Alkylamino Nitramines

Simen Antonsen, Marius Aursnes, Harrison Gallantree-Smith, Christian Dye and Yngve Stenstrøm

1. Spectral Data for Selected Compounds

3-Nitrooxazolidin-2-one (**8**)

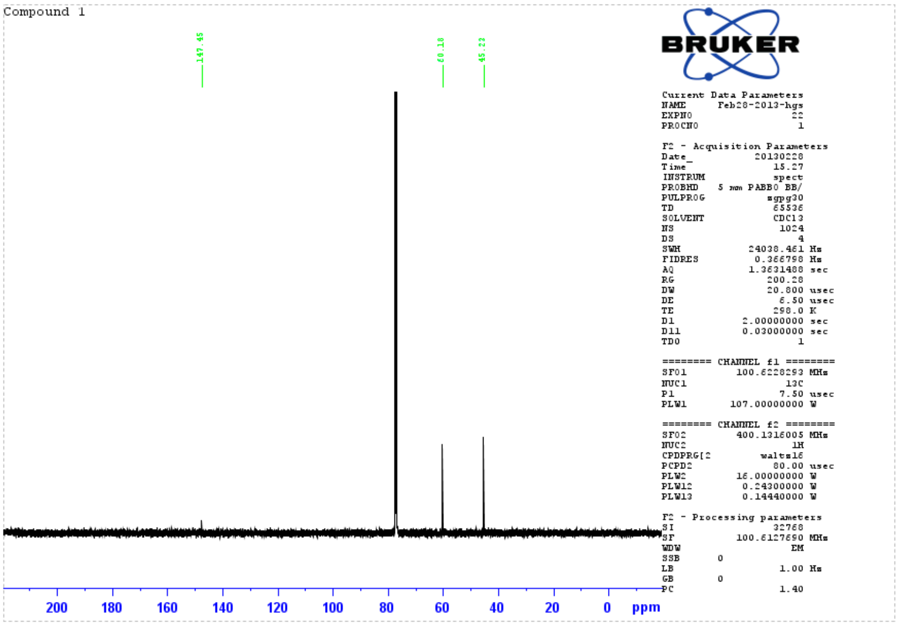

Figure S1. ^{13}C-NMR spectrum of 3-nitrooxazolidin-2-one (**8**).

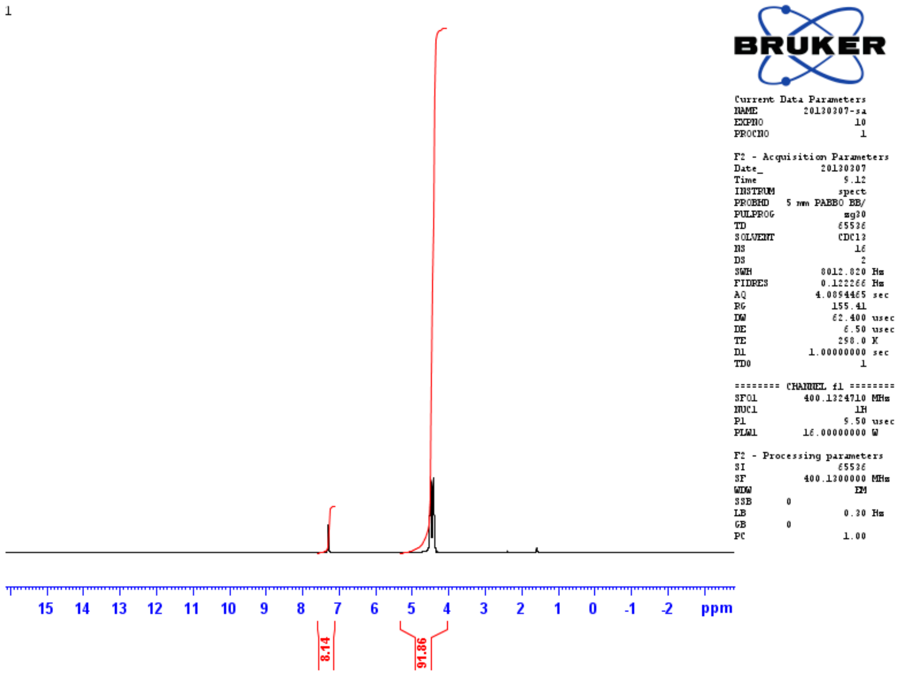

Figure S2. ^{1}H-NMR spectrum of 3-nitrooxazolidin-2-one (**8**).

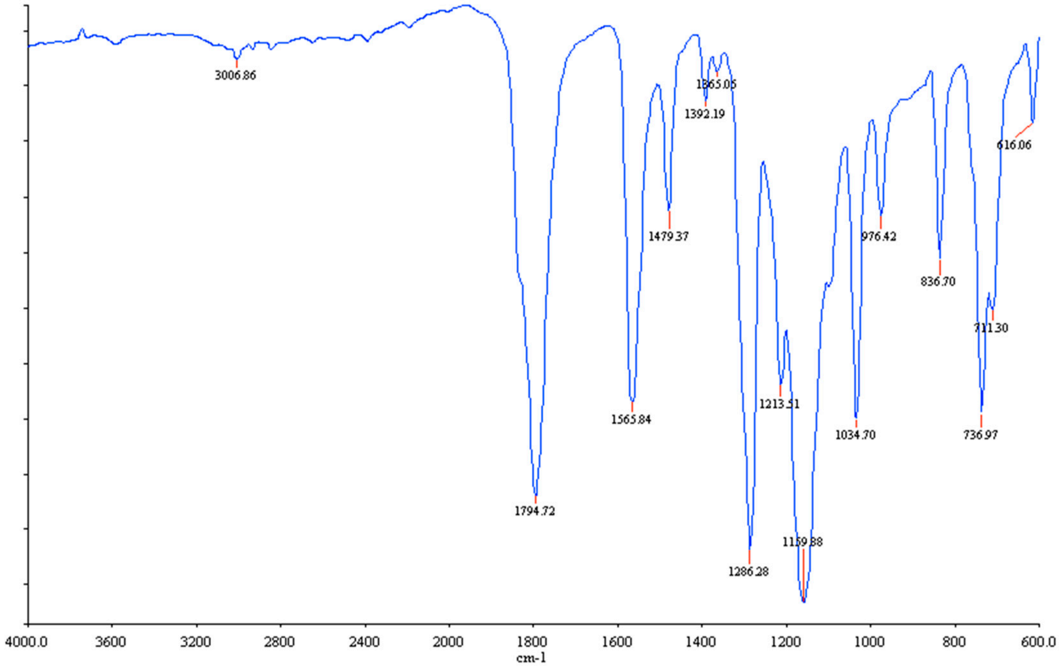

Figure S3. IR spectrum of 3-nitrooxazolidin-2-one (**8**).

4,4-Dimethyl-3-nitrooxazolidin-2-one (**9**)

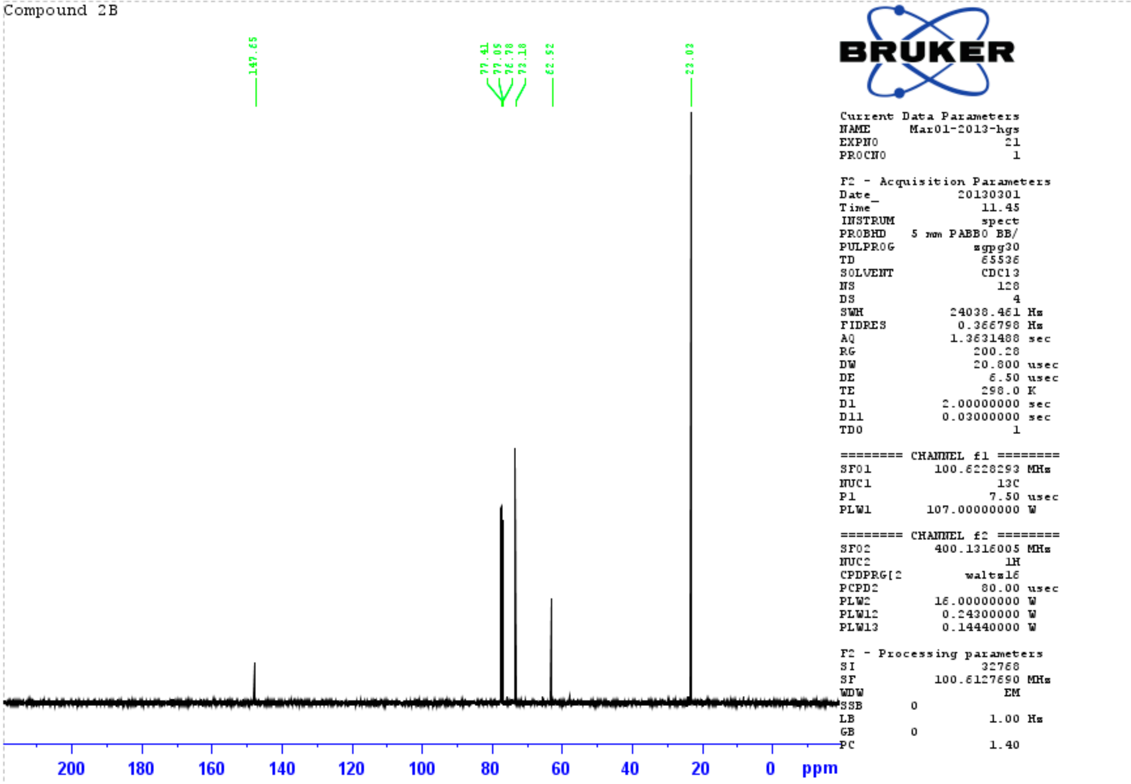

Figure S4. ^{13}C-NMR spectrum of 4,4-dimethyl-3-nitrooxazolidin-2-one (**9**).

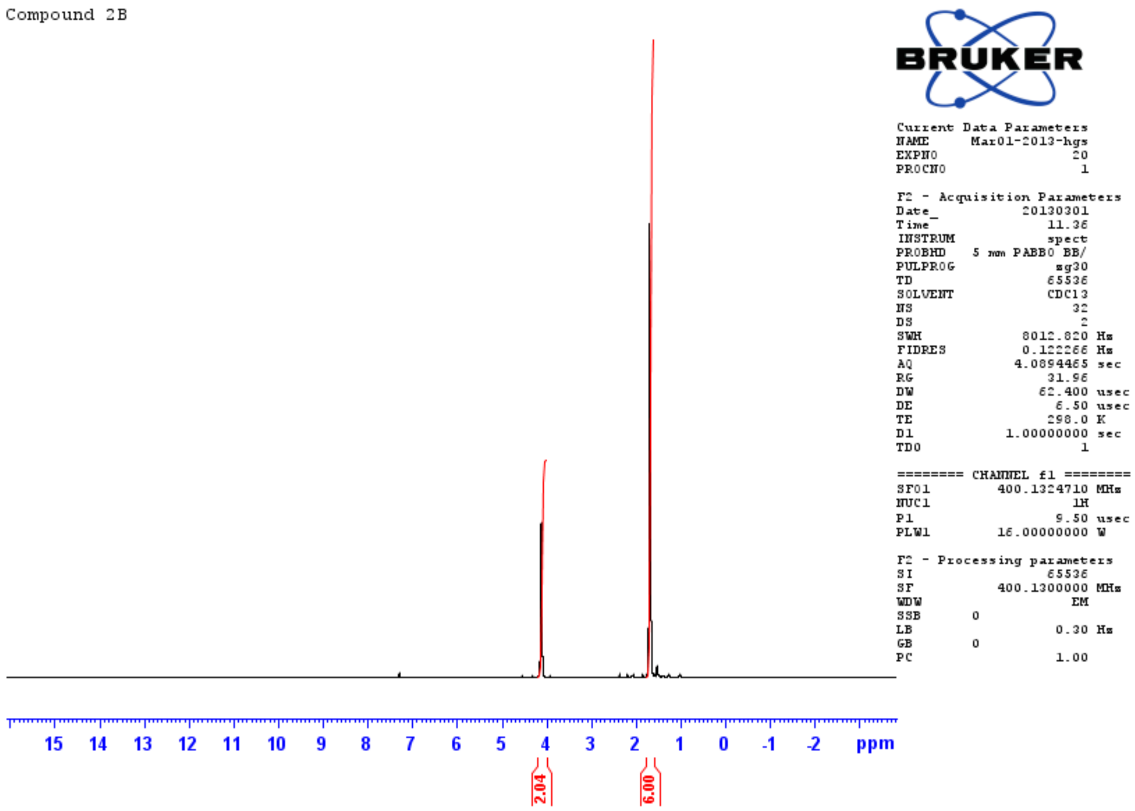

Figure S5. ^{1}H-NMR spectrum of of 4,4-dimethyl-3-nitrooxazolidin-2-one (**9**).

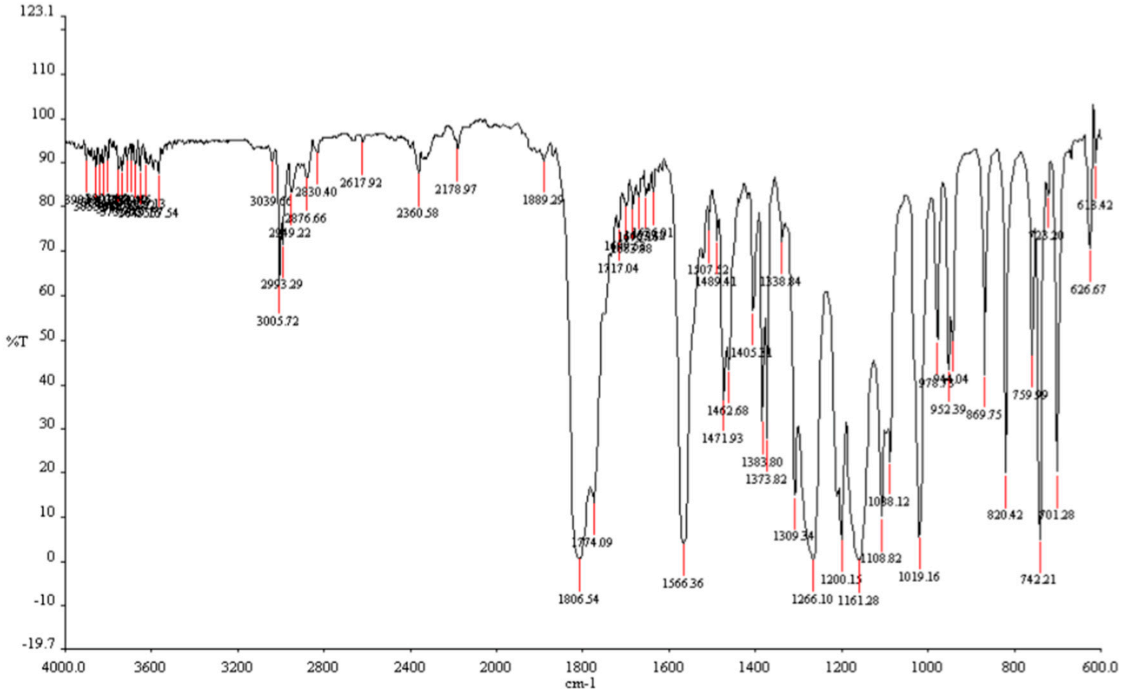

Figure S6. IR spectrum of 4,4-dimethyl-3-nitrooxazolidin-2-one (**9**).

3-Nitrobenzo[d]oxazol-2(3H)-one (**16**)

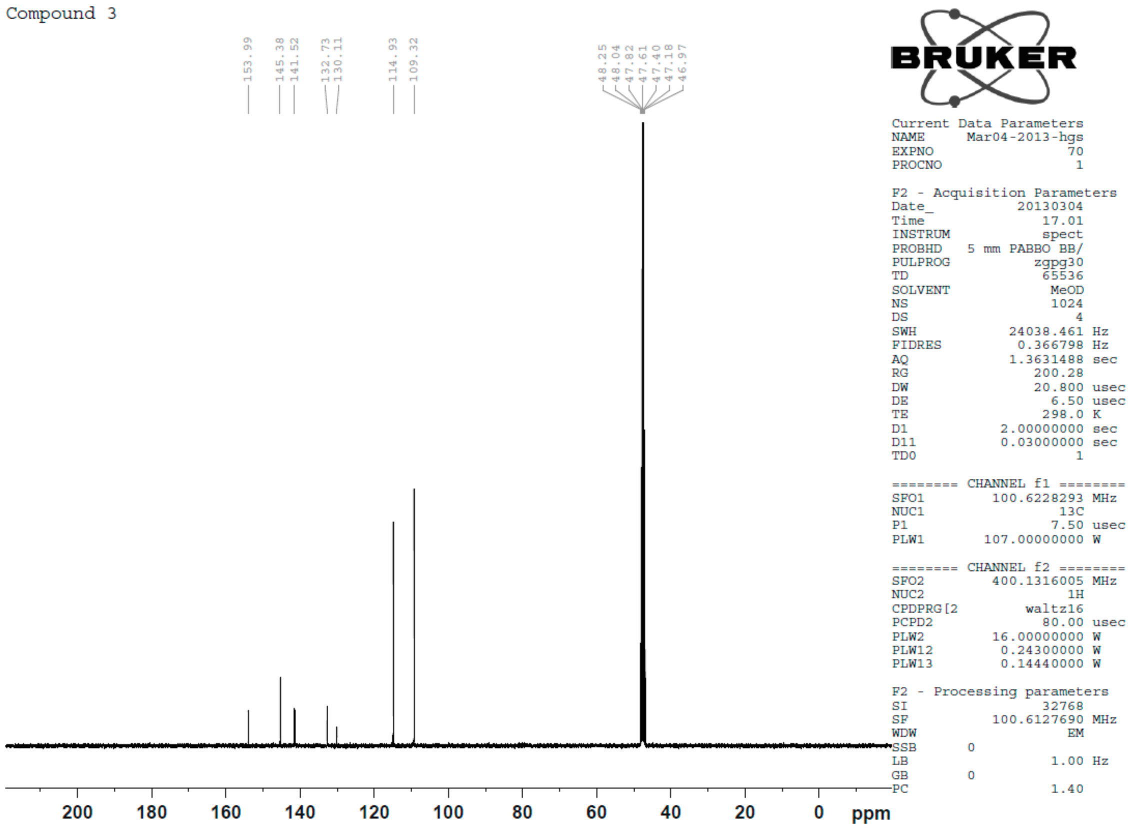

Figure S7. ^{13}C-NMR spectrum of 3-nitrobenzo[d]oxazol-2(3H)-one (**16**).

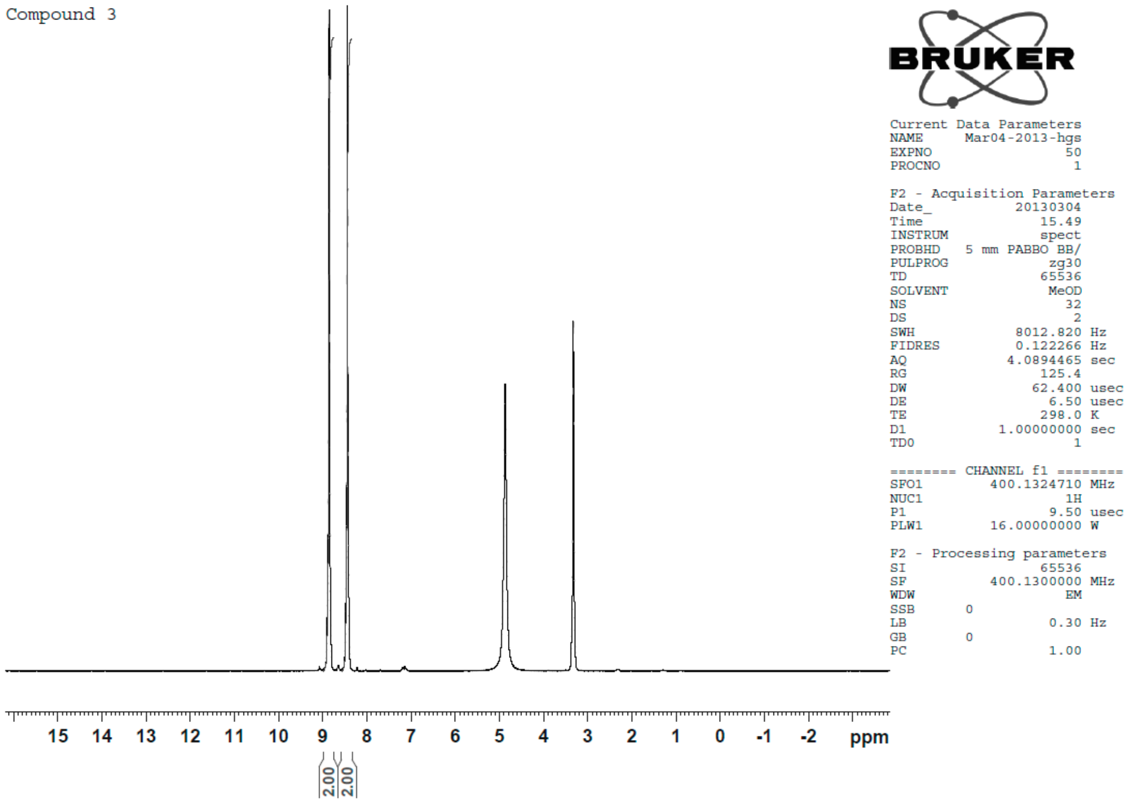

Figure S8. ^{1}H-NMR spectrum of 3-nitrobenzo[d]oxazol-2(3H)-one (**16**).

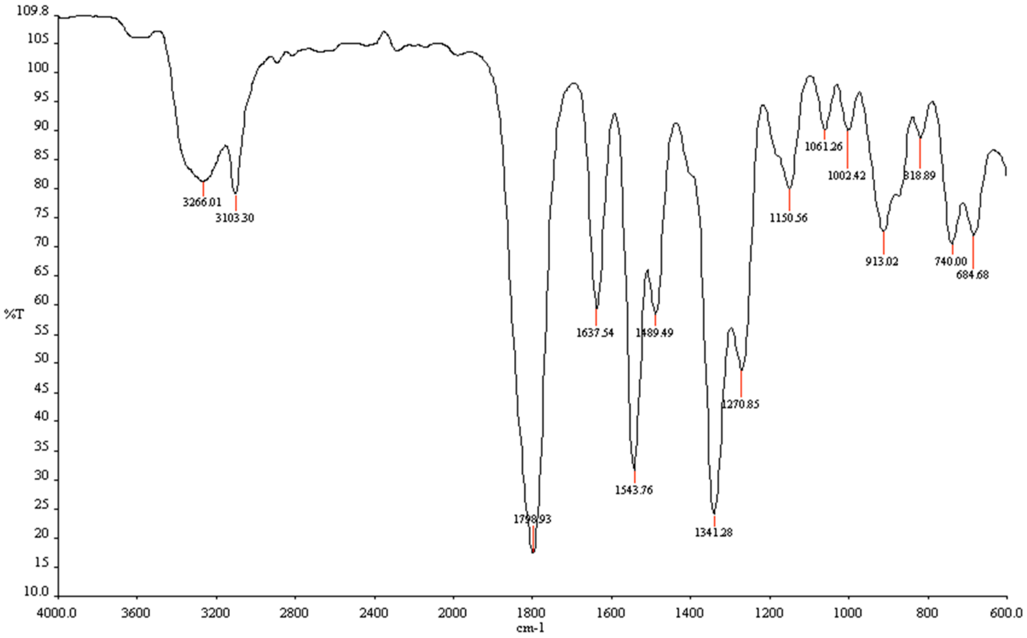

Figure S9. IR spectrum of 3-nitrobenzo[d]oxazol-2(3H)-one (**16**).

5-(Chloromethyl)-3-nitrooxazolidin-2-one (**17**)

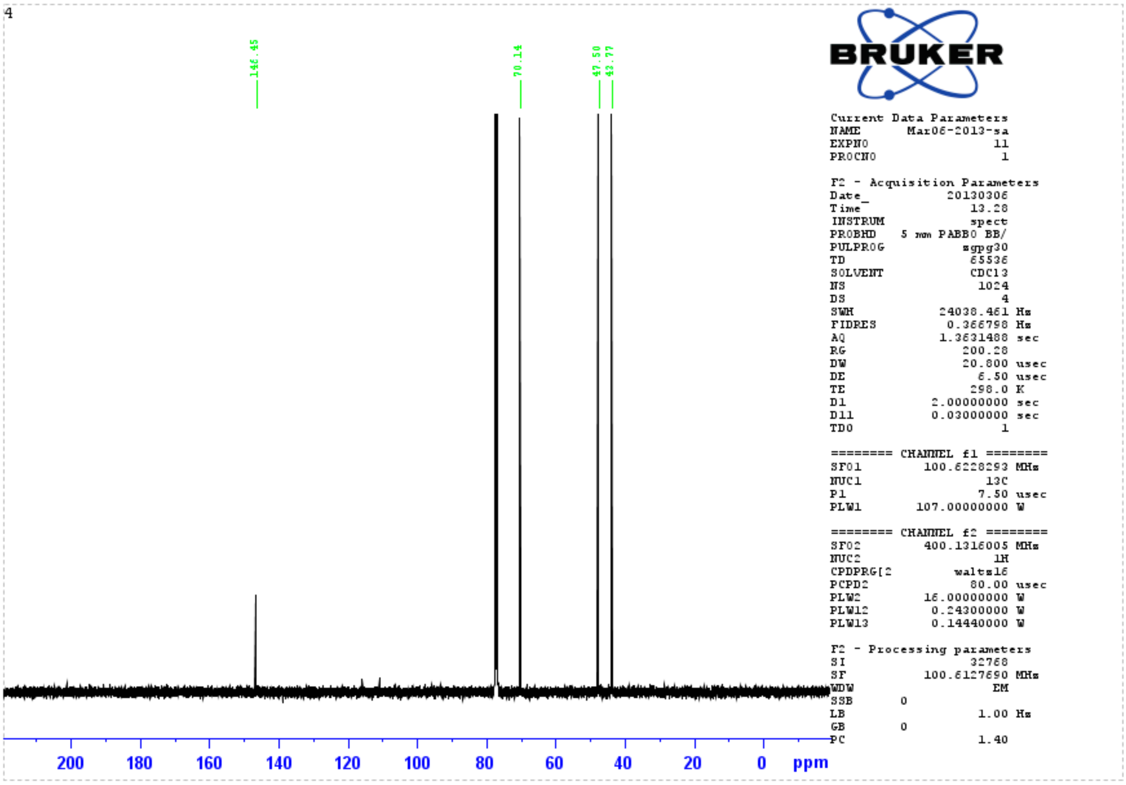

Figure S10. ^{13}C-NMR spectrum of 5-(chloromethyl)-3-nitrooxazolidin-2-one (**17**).

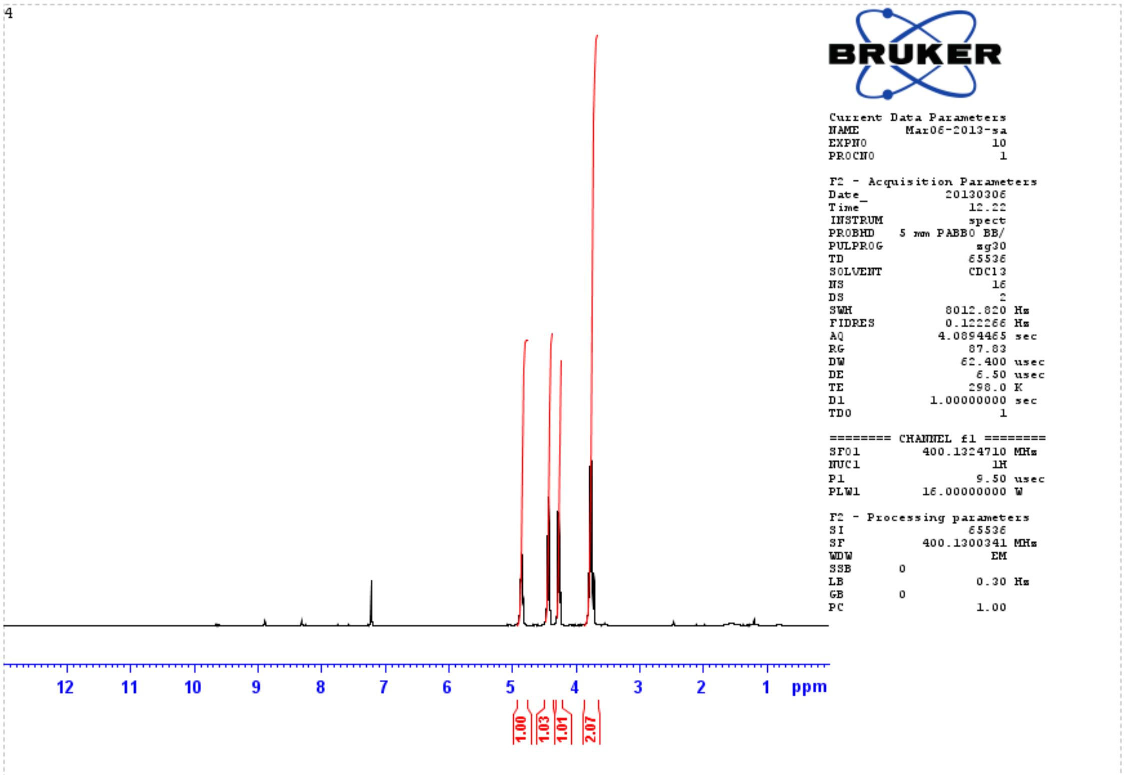

Figure S11. ^{1}H-NMR spectrum of 5-(chloromethyl)-3-nitrooxazolidin-2-one (**17**).

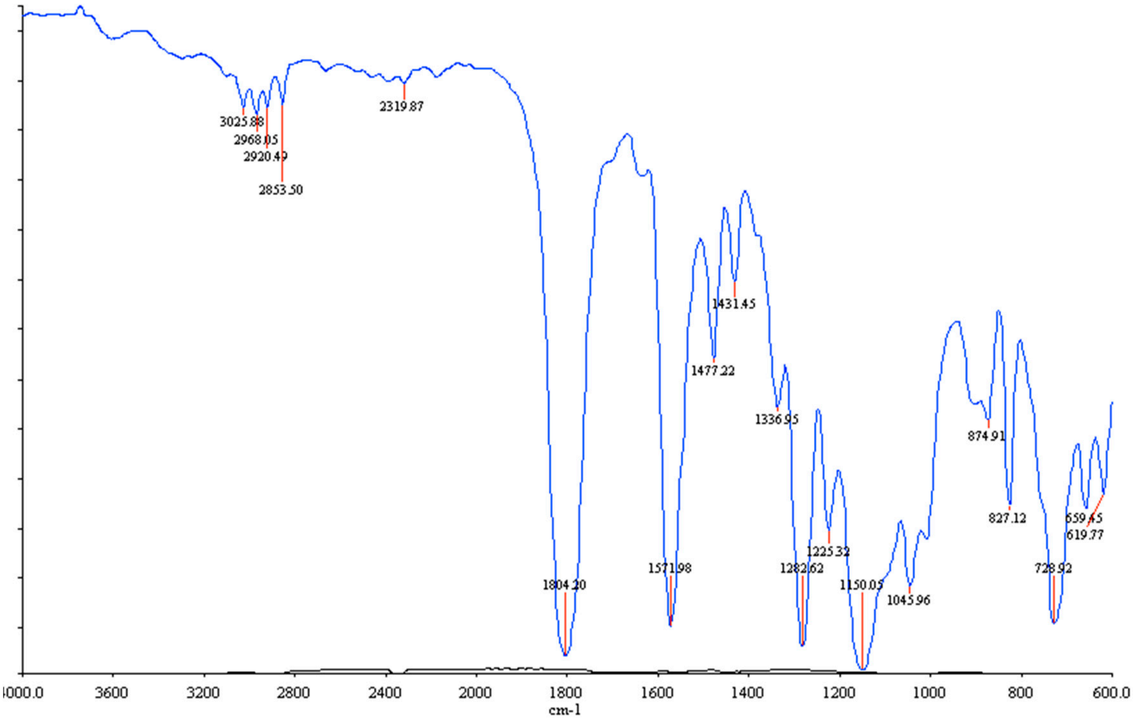

Figure S12. IR spectrum of 5-(chloromethyl)-3-nitrooxazolidin-2-one (**17**).

3-Nitro-1,3-oxazinan-2-one (**18**)

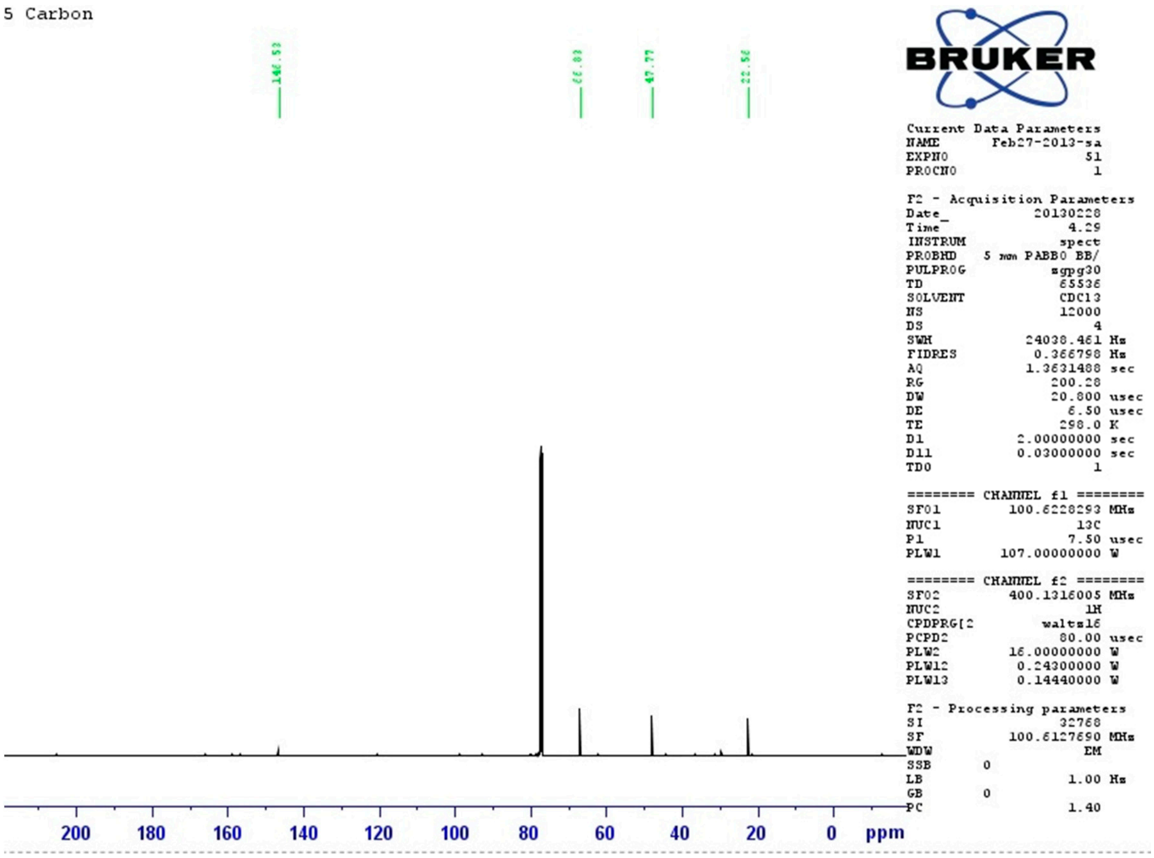

Figure S13. ^{13}C-NMR spectrum of 3-nitro-1,3-oxazinan-2-one (**18**).

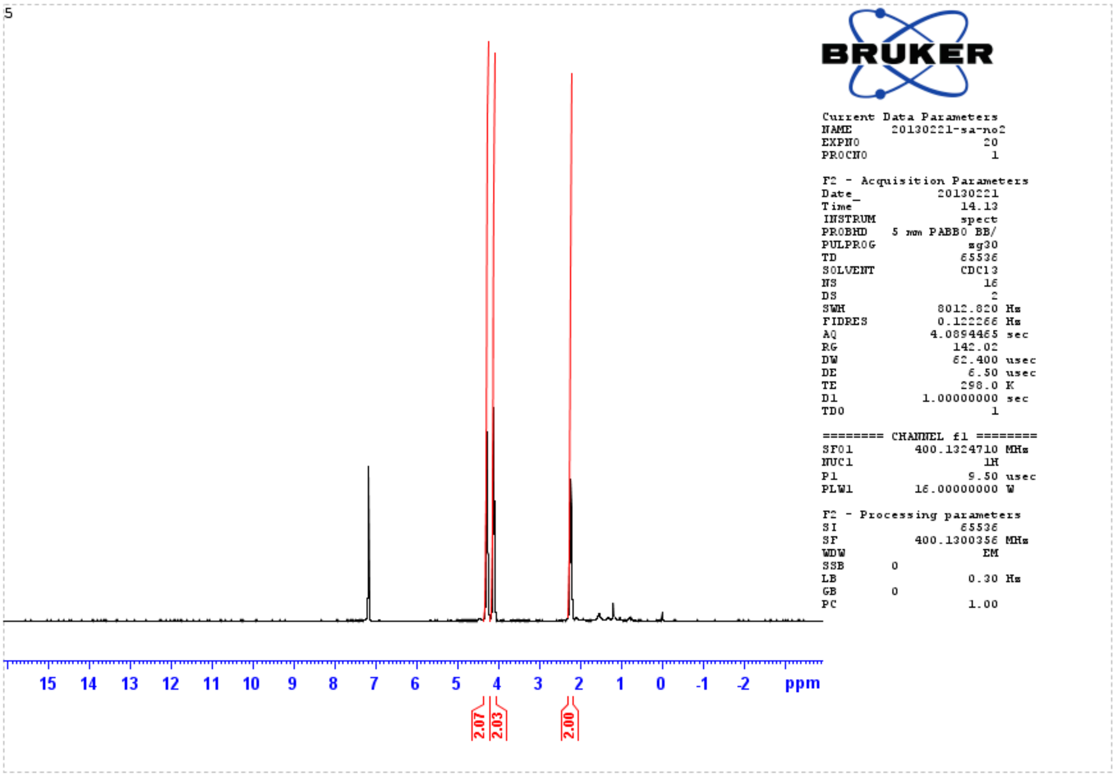

Figure S14. ^{1}H-NMR spectrum of ^{13}C-NMR spectrum of 3-nitro-1,3-oxazinan-2-one (**18**).

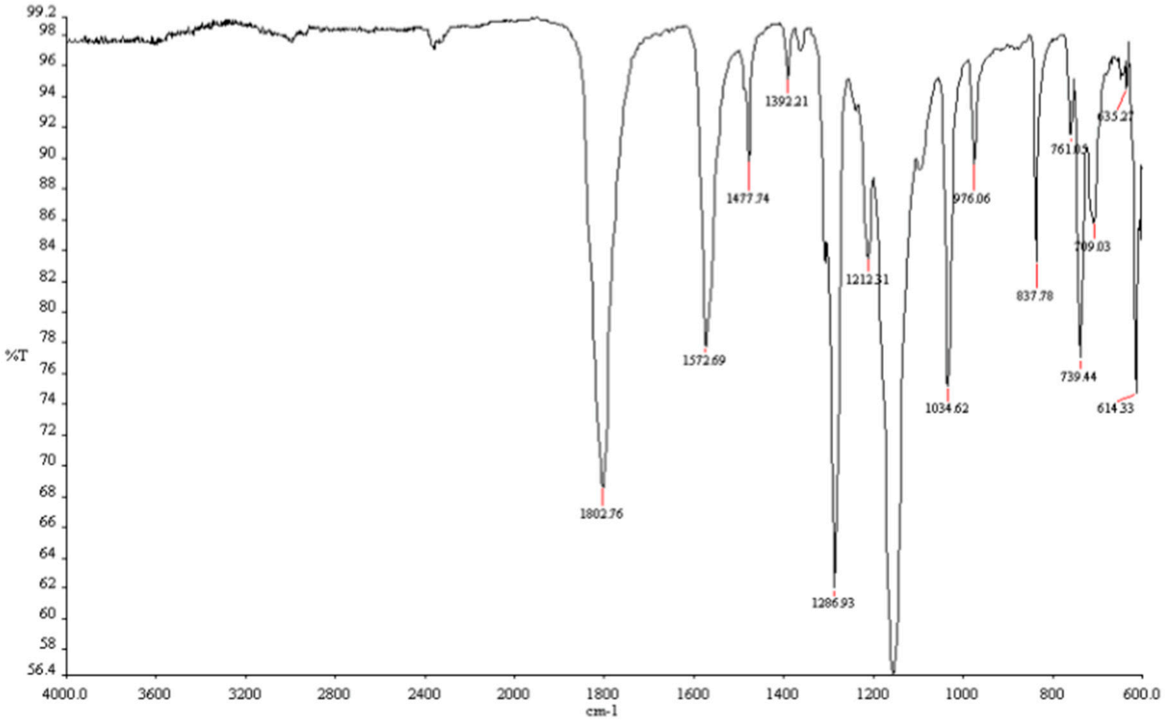

Figure S15. IR spectrum of ^{13}C-NMR spectrum of 3-nitro-1,3-oxazinan-2-one (**18**).

Methyl N-(tert-butoxycarbonyl)-N-nitro-alaninate (**19**)

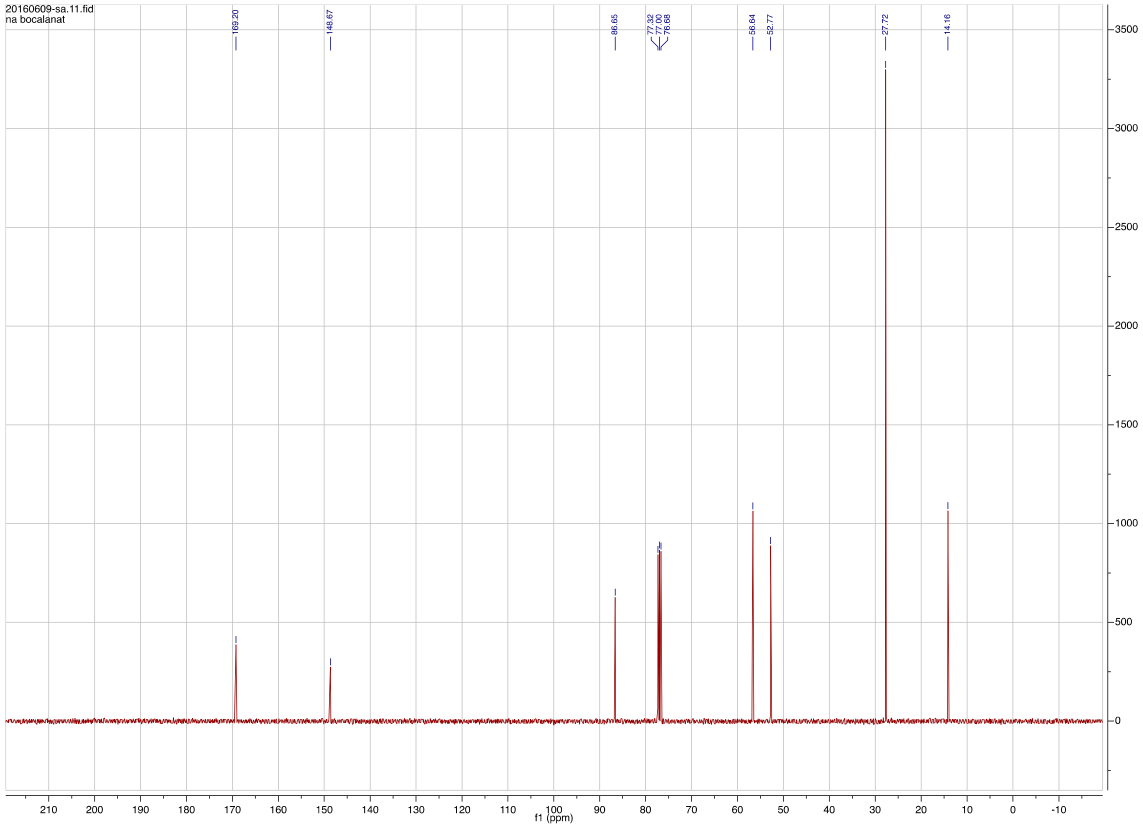

Figure S16. ^{13}C-NMR spectrum of Methyl *N*-(tert-butoxycarbonyl)-*N*-nitro-alaninate (**19**).

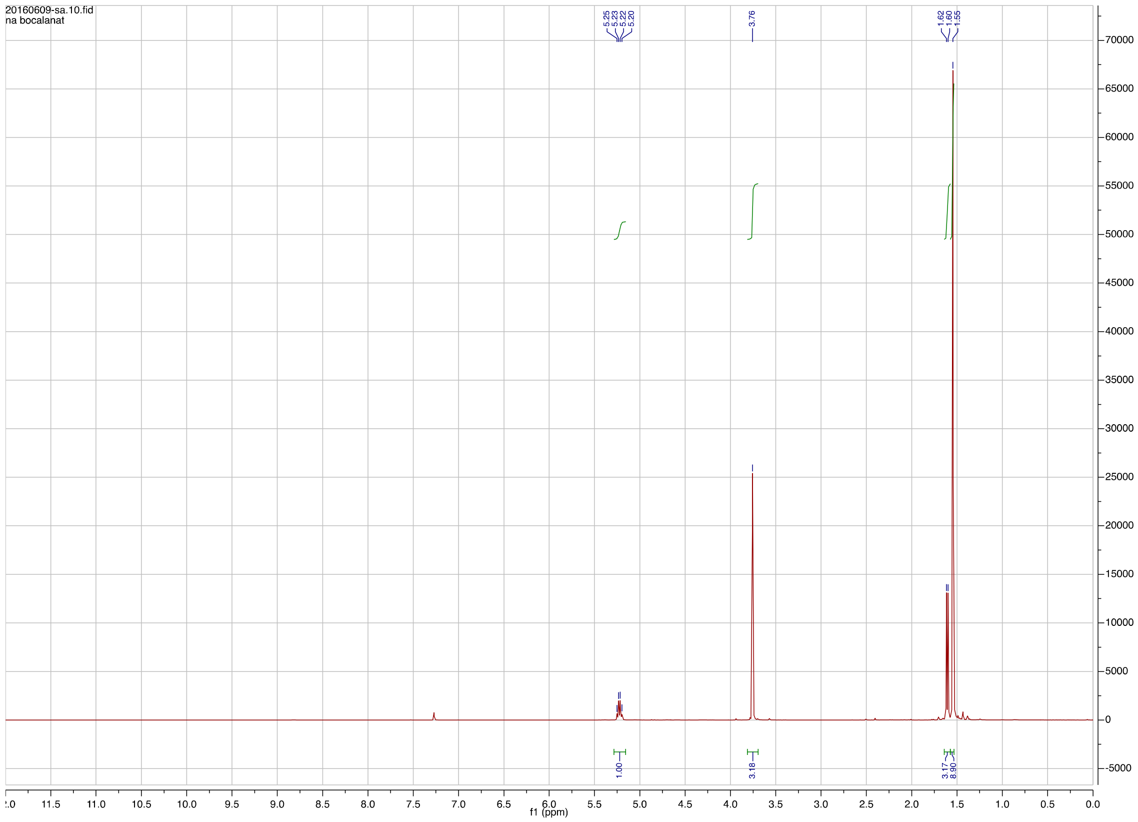

Figure S17. ^{1}H-NMR spectrum of Methyl *N*-(tert-butoxycarbonyl)-*N*-nitro-alaninate (**19**).

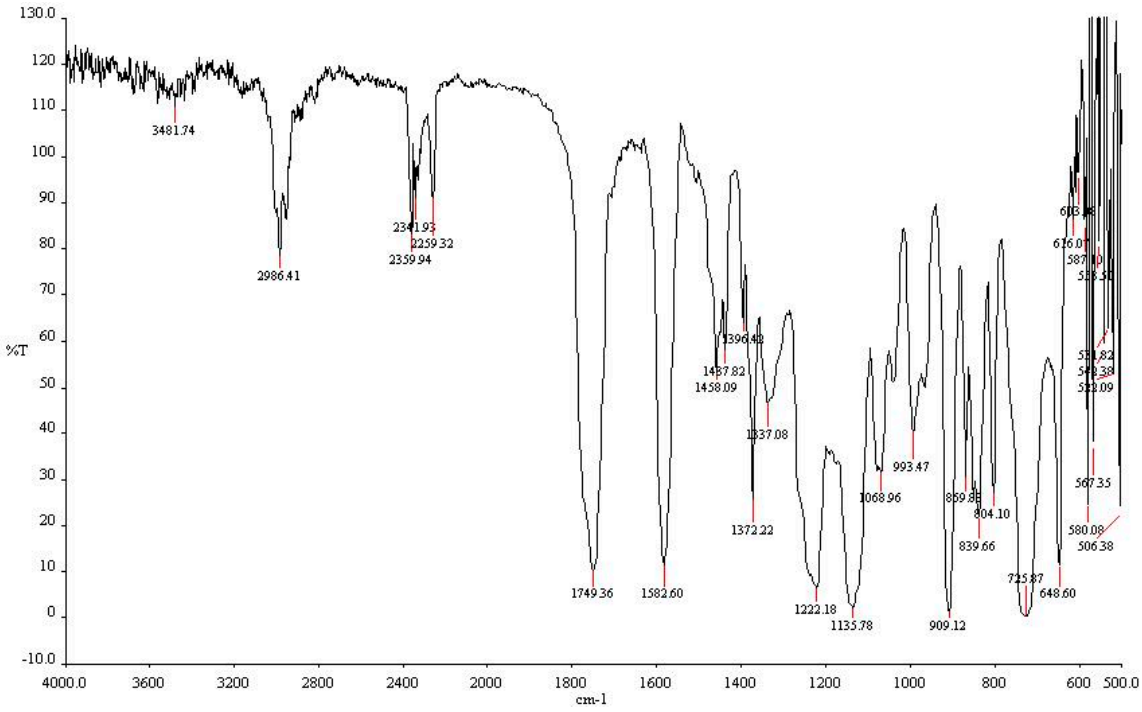

Figure S18. IR spectrum of Methyl *N*-(tert-butoxycarbonyl)-*N*-nitro-alaninate (**19**).

Table S1. Monitoring parameters for detection of nitramines with HPLC/UV/HRMS.

Analyte	Molecular Weight *	Monit. Ion $[M - H]^-$	Ionisation Mode
2-hydoxyethylnitramine	106.0378	105.0300	ES-
1-hydroxy-2-methylpropan-2-ylnitramine	134.0691	133.0613	ES-

* Monoisotopic.

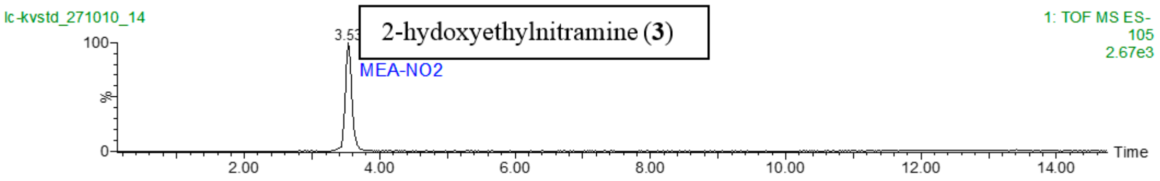

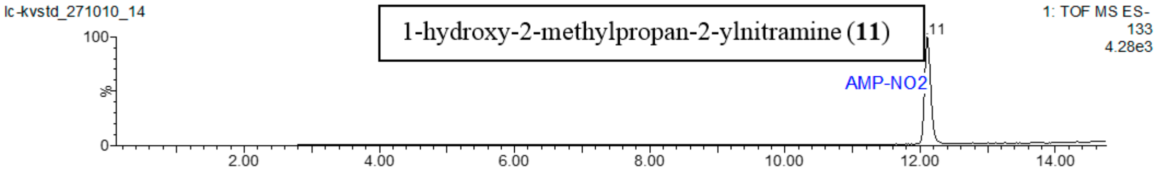

Figure S19. Separation of the nitramines by the HPLC column Waters Atlantis dC18, 3 µm, 2.1 × 150 mm, using a binary water/acetonitrile gradient.

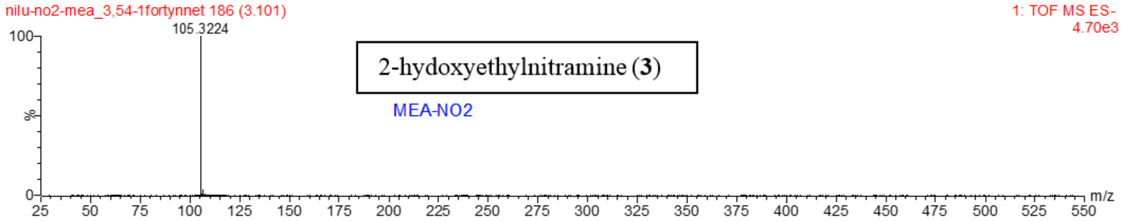

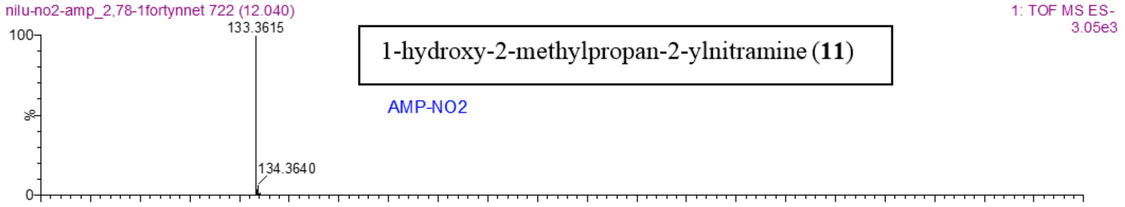

Figure S20. HRMS spectra of the ions $[M - H]^-$ of 2-hydoxyethylnitramine and 1-hydroxy-2-methylpropan-2-ylnitramine obtained by HPLC/HRMS.

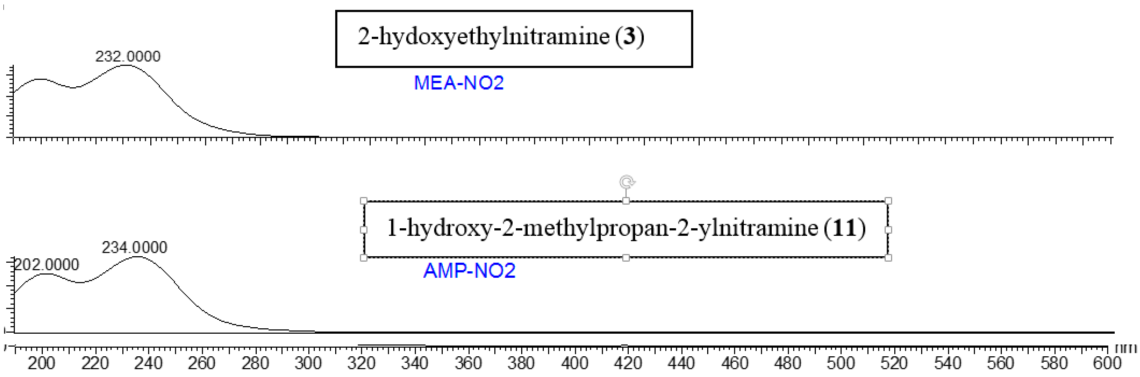

Figure S21. UV-spectra of the nitramines obtained by HPLC/UV.
